# Nematophilic bacteria associated with entomopathogenic nematodes and drug development of their biomolecules

**DOI:** 10.3389/fmicb.2022.993688

**Published:** 2022-09-15

**Authors:** Ryan Musumba Awori

**Affiliations:** ^1^Department of Biology, University of Nairobi, Nairobi, Kenya; ^2^Elakistos Biosciences, Nairobi, Kenya

**Keywords:** nematophilic bacteria, *Xenorhabdus* bacteria, *Photorhabdus* bacteria, entomopathogenic nematode (EPN), natural product (NP), pangenomics, drug development

## Abstract

The importance of *Xenorhabdus* and *Photorhabdus* symbionts to their respective *Steinernema* and *Heterorhabditis* nematode hosts is that they not only contribute to their entomopathogenicity but also to their fecundity through the production of small molecules. Thus, this mini-review gives a brief introductory overview of these nematophilic bacteria. Specifically, their type species, nematode hosts, and geographic region of isolations are tabulated. The use of nucleotide sequence-based techniques for their species delineation and how pangenomes can improve this are highlighted. Using the *Steinernema*–*Xenorhabdus* association as an example, the bacterium-nematode lifecycle is visualized with an emphasis on the role of bacterial biomolecules. Those currently in drug development are discussed, and two potential antimalarial lead compounds are highlighted. Thus, this mini-review tabulates forty-eight significant nematophilic bacteria and visualizes the ecological importance of their biomolecules. It further discusses three of these biomolecules that are currently in drug development. Through it, one is introduced to *Xenorhabdus* and *Photorhabdus* bacteria, their natural production of biomolecules in the nematode-bacterium lifecycle, and how these molecules are useful in developing novel therapies.

## Introduction

Nematophilic “nematode loving” bacteria are prokaryotes that are symbiotically associated with members of phylum *Nematoda*. Three such genera are *Xenorhabdus*, *Photorhabdus*, and *Serratia*, which are symbionts of *Steinernematidae*, *Heterorhabditidae*, and *Rhabditidae* members, respectively. Each of these three families of the order *Rhabditida* contain entomopathogenic species—all members of *Steinernema* and *Heterorhabditis*, all members of the Insectivora—group of *Oscheius*, and *Caenorhabditis briggsae*. However, it is their *Xenorhabdus*, *Photorhabdus*, or *Serratia* symbionts that contribute in large part to this trait through both septicemia and toxemia (Bowen et al., 1998; Ffrench-Constant and Waterfield, 2005; Abebe et al., 2011; Clarke, 2020). Hence, *Xenorhabdus, Photorhabdus*, and a few *Serratia* strains are also termed entomopathogenic bacteria. Whereas *Serratia* symbionts form associations with *Oscheius* and *Caenorhabditis* hosts, *Xenorhabdus* and *Photorhabdus* are more genus-specific associating only with *Steinernema* and *Heterorhabditis* hosts, respectively (Table 1).

**TABLE 1:** Nematophilic bacteria associated with entomopathogenic nematodes (EPNs).

Species	Nematode host of isolation	Geographic origin of nematode	Example of a bioactive molecule produced by the type strain
*Xenorhabdus beddingii* (Akhurst and Boemare, 1988)	*Steinernema longicaudum*	Tasmania, Australia (Akhurst, 1983)	Xefoampeptide (Tobias et al., 2017; Kegler and Bode, 2020)
*X. bovienii* (Akhurst and Boemare, 1988)	*S. affine*	Tasmania, Australia (Akhurst, 1983)	Xenocyloin (Proschak et al., 2014)
	*S. intermedium*		
	*S. kraussei*		
	*S. feltiae*		
*X. budapestensis* (Lengyel et al., 2005)	*S. bicornutum*	Szabadka, Serbia (Tallosi et al., 1995)	Bicornutin (Böszörményi et al., 2009)
*X*. *cabanillasii* (Tailliez et al., 2006)	*S. riobrave*	Weslaco, USA (Cabanillas et al., 1994)	Rhabdopeptide (Reimer et al., 2013)
*X. doucetiae* (Tailliez et al., 2006)	*S. diaprepesi*	Martinique, Caribbean (Fischer-Le Saux et al., 1998)	Xenorhabdin (Bode et al., 2015)
*X. eapokensis* (Kämpfer et al., 2017)	*S. eapokense*	Eapok, Vietnam (Phan et al., 2006)	GameXPeptide (Tobias et al., 2017; Shi et al., 2022)
X. *ehlersii* (Lengyel et al., 2005)	*S. serratum*	Shangdong, China (Qiu et al., 2004)	GameXPeptide (Tobias et al., 2017; Shi et al., 2022)
*X. griffiniae* (Tailliez et al., 2006)	*S. hermaphroditum*	Kamal, Indonesia (Stock et al., 2004)	
*X. hominickii* (Tailliez et al., 2006)	*S. karii*	Kirinyaga, Kenya (Waturu et al., 1997)	Fabclavine (Wenski et al., 2020)
	*S. monticolum*		
*X. indica* (Somvanshi et al., 2006)	*S. thermophilum*	New Delhi, India (Sudershan and Singh, 2000)	Taxlllaid (Kronenwerth et al., 2014)
*X. innexi* (Lengyel et al., 2005)	*S. scapterisci*	Rivera, Uruguay (Nguyen and Smart, 1990)	Rhabdopeptide/xenortide-like peptide (Zhao L. et al., 2018)
*X. japonica* (Nishimura et al., 1994)	*S. kushidai*	Hamakita, Japan (Nishimura et al., 1994)	Lipocitide (Shi et al., 2022)
*X. ishibashii* (Kuwata et al., 2013)	*S. aciari*	Haimen, China (Qiu et al., 2005)	Xenorhabdin (McInerney et al., 1991; Bode et al., 2015; Tobias et al., 2017)
*X. lircayensis* (Castaneda-Alvarez et al., 2021)	*S. unicornum*	Altos de Lircay, Chile (Castaneda-Alvarez et al., 2021)	
*X. khoisanae* (Ferreira et al., 2013)	*S. khoisanae*	Villiersdorp, South Africa (Malan et al., 2006)	
*X. koppenhoeferi* (Tailliez et al., 2006)	*S. scarabaei*	New Jersey, USA (Stock and Koppenhöfer, 2003)	
*X. kozodoii* (Tailliez et al., 2006)	*S. arenarium*	Voronezh, Russia (Artyukhovsky, 1997)	Xenocoumacin (Park et al., 2009; Tobias et al., 2017)
	*S. apuliae*		
*X. magdalenensis* (Tailliez et al., 2012)	*S. australe*	Isla Magdalena, Chile (Edgington et al., 2009)	
*X. mauleonii* (Tailliez et al., 2006)	*Steinernema* sp.	St. Vincent, Caribbean (Fischer-Le Saux et al., 1998)	Xenoamicin (Zhou et al., 2013)
*X. miraniensis* (Tailliez et al., 2006)	*Steinernema* sp.	Mirani, Australia (Akhurst and Boemare, 1988)	Ambactin (Schimming et al., 2014)
*X. nematophila* (Akhurst and Boemare, 1988)	*S. carpocapsae*	Virginia, USA (Poinar et al., 1972)	Rhabduscin (Eugenia Nuñez-Valdez et al., 2019)
*X*. *poinarii* (Akhurst and Boemare, 1988)	*S. glaseri*	North Carolina, USA (Poinar, 1978)	
	*S. cubanum*		
*X. romanii* (Tailliez et al., 2006)	*S. puertoricense*	Puerto Rico, USA (Román and Figueroa, 1994)	
*X. stockiae* (Tailliez et al., 2006)	*S. siamkayai*	Lohmsak, Thailand (Stock, 1998)	GameXPeptide (Tobias et al., 2017; Shi et al., 2022)
*X. szentirmaii* (Lengyel et al., 2005)	*S. rarum*	Cordoba, Argentina (Aguera de Doucet, 1986)	Szentiamide (Ohlendorf et al., 2011)
*X. thuongxuanensis* (Kämpfer et al., 2017)	*S. sangi*	Thuongxuan, Vietnam (Phan et al., 2001)	GameXPeptide (Tobias et al., 2017; Shi et al., 2022)
*X. vietnamensis* (Tailliez et al., 2010)	*S. sangi*	Xuanmy, Vietnam (Phan et al., 2001)	Benzobactin A (Shi et al., 2022)
*Serratia nematodiphila* (Zhang et al., 2009)	*Oscheius chongmingensis*	Chongming Islands, China (Zhang et al., 2009)	
*S. marcescens* (Torres-Barragan et al., 2011)	*O. carolinensis*	Raleigh, USA (Ye et al., 2010)	
	*O. safricana*	Northwest Province, South Africa (Serepa-Dlamini and Gray, 2018)	
*Serratia* sp. strain TEL (Lephoto Tiisetso et al., 2015)	*O. basothovii*	Suikerbosrand Nature Reserve, South Africa (Lephoto Tiisetso et al., 2015)	
*Serratia* sp. strain N19 (Zhou et al., 2017)	*O. microvilli*	Chongming Island, China (Zhou et al., 2017)	
*Serratia* sp. strain SCBI (Abebe et al., 2011)	*Caenorhabditis briggsae*	Mpumalanga Province, South Africa (Abebe et al., 2010)	
*Photorhabdus aegyptia* (Machado et al., 2021a)	*Heterorhabditis bacteriophora*	Egypt (Hussein and El-Souud, 2006)	Piscibactin (Shi et al., 2022)
*P. akhurstii* (Machado et al., 2018)	*H. indica*	Grande Terre, Guadeloupe Islands (Fischer-Le Saux et al., 1998)	
*P. asymbiotica* (Fischer-Le Saux et al., 1999)	Unknown	San Antonio, USA (Farmer et al., 1989)	
*P. australis* (Machado et al., 2018)	*H. gerrardi*	Victoria, Australia (Peel et al., 1999; Plichta et al., 2009)	Glidobactin (Tobias et al., 2017)
*P. bodei* (Machado et al., 2018)	*H. beicherriana*	Liaoning Province, China (Machado et al., 2018)	Photoxenobactin (Shi et al., 2022)
*P. caribbeanensis* (Machado et al., 2018)	*H. bacteriophora*	Basse Terre, Guadeloupe Islands (Fischer-Le Saux et al., 1998)	
*P. cinerea* (Machado et al., 2018)	*H. downesi*	Ásotthalom, Hungary (Tóth and Lakatos, 2008)	
*P. hainanensis* (Machado et al., 2018)	*Heterorhabditis* sp.	Hainan Island, China (Akhurst, 1987)	
*P. heterorhabditis* (Ferreira et al., 2014)	*H. zealandica*	Brits, South Africa (Mothupi, 2016)	
*P. hindustanensis* (Machado et al., 2021b)	*Heterorhabditis* sp.	Meghalaya, India (Ganguly et al., 2010)	
*P. kleinii* (Machado et al., 2018)	*H. georgiana*	Ohio, USA (An and Grewal, 2011)	
*P. kayaii* (Machado et al., 2018)	*H. bacteriophora*	Aksaray, Turkey (Hazir et al., 2003)	
*P. khanii* (Machado et al., 2018)	*H. bacteriophora*	Clayton, USA (Khan et al., 1976)	
*P. laumondii* (Machado et al., 2018)	*H. bacteriophora*	Trindad, Trindad and Tobago (Fischer-Le Saux et al., 1998)	Makes Caterpillar Floppy toxin (Daborn et al., 2002)
*P. luminescens* (Boemare et al., 1993)	*H. bacteriophora*	Brecon, Australia (Thomas and Poinar, 1979)	3,5-dihydroxy-4-isopropylstilbene (Hu et al., 1997)
*P. namnaonensis* (Machado et al., 2018)	*H. baujardi*	Nam Nao, Thailand (Glaeser et al., 2017)	3-isopropyl-4-oxo-2-oxetanecarboxylic acid (Shi et al., 2022)
*P. noenieputensis* (Machado et al., 2018)	*H. noenieputensis*	Nelspruit, South Africa (Malan et al., 2011)	
*P. stackebrandtii* (Machado et al., 2018)	*H. bacteriophora*	Atwood, USA (Grewal et al., 2002)	
*P. tasmaniensis* (Machado et al., 2018)	*H. zealandica*	Nicholls Rivulet, Australia (Akhurst, 1987)	
*P. temperata* (Fischer-Le Saux et al., 1999)	*H. megidis*	Nachodka, Russia (Akhurst, 1987)	
*P. thracensis* (Machado et al., 2018)	*H. bacteriaphora*	Kirklareli, Turkey (Hazir et al., 2003)	GameXPeptide (Tobias et al., 2017; Shi et al., 2022)

Apart from *Xenorhabdus* and *Serratia*, other entomopathogenic bacterial symbionts such as *Pseudomonas* sp. (Ogier et al., 2020) and *Alcaligenes* sp. (Shan et al., 2019) associate with *Steinernema* and *Oscheius* nematodes, respectively.

This classification of *Serratia* as nematophilic bacteria has caveats. Not all species in the *Serratia* genus are nematode symbionts (Grimont and Grimont, 2006), and only a few *Oscheius-Serratia*/*Caenorhabditis-Serratia* associations are known (Table 1). Moreover, for some of these associations, *Serratia* were only facultative symbionts—*Serratia* sp. strain SCBI and *Serratia marcescens* from *C. briggsae* and *Oscheius carolinensis*, respectively (Abebe et al., 2011; Torres-Barragan et al., 2011). Conversely, except one, all characterized *Xenorhabdus* and *Photorhabdus* species are natural nematode intestinal symbionts (Table 1). Thus, this mini-review focused on *Xenorhabdus* and *Photorhabdus* as nematophilic bacteria.

## *Xenorhabdus* and *Photorhabdus* bacteria

*Xenorhabdus* and *Photorhabdus* are both gram-negative, rod-shaped, peritrichously flagellated, facultative anaerobes of the family *Morganellaceae*, order *Enterobacterales*, and class *Gammaproteobacteria* (Adeolu et al., 2016). They are uniquely characterized by not only having primary and secondary variants but also an endosymbiosis with entomopathogenic nematodes (EPNs). Other distinguishing traits include *Photorhabdus* as the only terrestrial bioluminescent bacterium genus and *Xenorhabdus* as the only member of *Enterobacterales* that does not produce catalase (Boemare and Akhurst, 2006). Despite this taxonomic relatedness, the similar ecological niche of the two is more due to convergent evolution (Chaston et al., 2011). Twenty-seven *Xenorhabdus* species that were isolated from twenty-seven steinernematids have been described to date (Table 1). However, 100 *Steinernema* species have been characterized (Bhat et al., 2020) highlighting that at most—because of species with more than one nematode host (Table 1)—63 novel *Xenorhabdus* species could be added to the genus from these respective under-investigated yet described steinernematids. This prediction can be mathematically supported by determining whether the *Xenorhabdus* pangenome is open (Medini et al., 2020).

Nucleotide sequence-based techniques are not only the gold standard for prokaryotic species delineation (Chun et al., 2018) but are also useful for either identification of new isolates or emendation of already described taxon. For example, *Xenorhabdus* sp. strain BMMCB was described as an *Xenorhabdus. griffiniae* species (Mothupi et al., 2015), but we (Awori et al., 2017) demonstrated that its nucleotide identities values for the recombinase A (*recA*), phosphoserine transferase (*serC*), and small subunit ribosomal ribonucleic acid (rRNA) (SSU) genes, with those of the type species, were below the accepted threshold for conspecific strains—97% for protein-coding genes (Tailliez et al., 2010) and 98.7% for SSU gene (Kim et al., 2014).

Two powerful nucleotide sequence-based techniques are average nucleotide identities (ANI) and digital DNA–DNA hybridization (dDDH), which both delineate species by calculating how related two genomes are. The thresholds for conspecific strains are >95% (Richter and Rosselló-Móra, 2009) and >70% (Auch et al., 2010) for ANI and dDDH, respectively. Both were used to reclassify *Photorhabdus* species (Machado et al., 2018). However, strains S8-52, S9-53, and S10-54 identified as *Photorhabdus kleinii* had ANI values of 96.7% with the *Photorhabdus bodei* type strain and *Photorhabdus temperata* Meg1 had ANI values of 96.3% with the *Photorhabdus thracensis* type strain demonstrating the difficulty in delineating species of *Photorhabdus* (Fischer-Le Saux et al., 1998; Tailliez et al., 2010; Machado et al., 2021b) even with these nucleotide-based thresholds (Bobay, 2020). Thus, the use of pangenome analysis for species delineations—as was done in the *Prochlorococcus* genus (Moldovan and Gelfand, 2018)—is recommended for *Photorhabdus* systematics when sufficient genome sequences—at least five per species (Medini et al., 2020)—are available.

## The nematode-bacterium lifecycle and bacterial biomolecules

**FIGURE 1:**
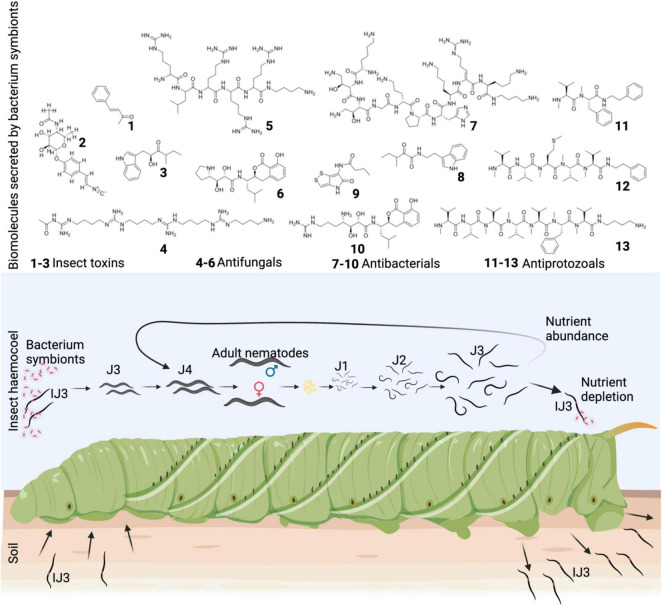
*Xenorhabdus-Steinernema* lifecycle and selected biomolecules that contribute toward nematode fecundity. Free-living infective third-stage juvenile (IJ3) nematodes seek out insects and gain entry through natural openings such as spiracles, and once within the hemocoel, nematodes exit their non-feeding state and release *Xenorhabdus* gut symbionts. The bacteria secrete a range of biomolecules (1–13) that increase the fecundity of the nematodes. Nematodes go through complete lifecycles thus increasing their numbers and upon depletion of nutrients each J3 re-associates with a few *Xenorhabdus* bacteria and exit the insect cadaver as an IJ3. J1, J2, J3, and J4 = first, second, third, and fourth larval stage juvenile nematodes, respectively. Benzylacetone (1), rhabduscin (2), xenocyloin (3), cabanillasin (4), biocornutin (5), xenocoumacin 2 (6), odilorhabdin (7), nematophin (8), xenorhabdin (9), xenocoumacin 1 (10), xenortide (11), rhabdopeptide (12), and rhabdopeptide/xenortide-like peptides (13) were created in biorender.com.

The nematode-bacterium lifecycle begins with soil-dwelling infective third larval stage juvenile nematode (IJ3) preying on an insect (Figure 1). Anatomically, IJ3 are third larval stage juvenile nematodes (J3) with a retained second larval stage cuticle that seals both mouth and anus rendering the nematodes into a non-feeding, developmentally arrested, and perennation-like stage (Poinar and Leutenegger, 1968). Steinernematids IJ3 infect an insect only through natural openings, whereas heterorhabditis can additionally gain entry by piercing into the hemocoel using a bursa (Bedding and Molyneux, 1982). Once within, the IJ3s undergo “recovery” (Clarke, 2020) whereby they shed their second larval stage cuticle and release into the hemocoel, their gut bacterial symbionts. For *Steinernema*, *Xenorhabdus* would have been previously localized in a receptacle (Stilwell et al., 2018) at the anterior gut whereas, in *Heterorhabditis, Photorhabdus* would have previously lined the entire gut (Waterfield et al., 2009). Detection of L-proline concentrations >4.8 mM in insect hemolymph triggers an upregulated bacterial secretion of specialized metabolites of various ecological functions (Crawford et al., 2010).

Despite the following grouping of biomolecules from both *Xenorhabdus* and *Photorhabdus* according to the similarity of ecological function, their biosynthesis is species-specific. The first grouping is insecticidal toxins, and these can be divided into insect immune suppressors *via* inhibition of phenoloxidase pathway:-1,2-benzene dicarboxylic acid (PA) (Ullah et al., 2014), benzylideneacetone (BZA) (Song et al., 2011), rhabduscin (Crawford et al., 2012; Eugenia Nuñez-Valdez et al., 2019), and 1,3-dihydroxy-2-(isopropyl)-5-(2-phenylethenyl)benzene (Eleftherianos et al., 2007); hemocyte pore-forming complexes: *Xenorhabdus* particulate toxins (Xpt) (Sheets et al., 2011), toxin complex toxins (Tc) (Blackburn et al., 1998), and *Xenorhabdus* α-xenorhabdolysin toxins (Xax) (Vigneux et al., 2007); apoptosis inducers: make caterpillar floppy toxins (Mcf) (Daborn et al., 2002; Dowling et al., 2004) and PaTox toxins (Jank et al., 2016); and those with yet unknown modes of action: PirAB (Yang et al., 2017) and xenocyloin (Proschak et al., 2014). Another ecological function of secreted metabolites is bioconversion by enzymes such as lipases, proteases, amylases, and proteases—their respective genes are enriched in *Xenorhabdus* and *Photorhabdus* genomes (Chaston et al., 2011)—creating a rich nutrient pool. To defend this from colonization by microbial competitors, a broad spectrum of antimicrobials is produced. These include antifungals: biocornutin (Böszörményi et al., 2009), cabanillasin (Houard et al., 2013), EP-19, GP-20 (Xiao et al., 2012), and xenocoumacin (Yang et al., 2011); antibacterials: darobactin (Imai et al., 2019), xenematide (Lang et al., 2008), photoditritide (Zhao et al., 2019), xenobactin (Grundmann et al., 2013), odilorhabdins (Pantel et al., 2018), xenorhabdin (McInerney et al., 1991), and PAX peptides (Gualtieri et al., 2009); antiprotozoals: phototemtide (Zhao et al., 2020), szentiamide (Ohlendorf et al., 2011), chaiyaphumins (Grundmann et al., 2014), rhabdopeptide/xenortide-like peptides (RXP) (Zhao L. et al., 2018), xenortide (Reimer et al., 2014), xenoamicin (Zhou et al., 2013), and ambactin (Schimming et al., 2014); and cytotoxic agents: fabclavines (Wenski et al., 2020) and phenylethylamine (PEA) derivatives (Proschak et al., 2011).

Recovered IJ3s leverage this nutrient-filled, enclosed environment to molt to fourth larval stage juvenile nematodes (J4) and then adults (Figure 1) that lay eggs after mating in the case of all steinernematids except *Steinernema hermaphroditum*—these species lay eggs without mating due to their hermaphroditic nature. This is like the androdioecious heterorhabditis whose adult females are also self-fertilized. Uniquely, *Heterorhabditis* adult females lay eggs into their uterus which hatch and develop into first larval stage juvenile nematodes (J1) through *endotokia matricida* (Clarke, 2020). Newly hatched EPNs molt from J1 through to J4 and then adults, which mate and lay eggs thus beginning another lifecycle. This continues until nutrients are depleted (Figure 1). Notably, infected cadavers are themselves protected from consumption by non-microbial competitors such as ants by the bacterial production of scavenger deterring factors (Zhou et al., 2002; Gulcu et al., 2012).

Upon nutrient depletion, J3 nematodes commence transformation to IJ3s by reassociating with bacterial symbionts (Figure 1)—this can be as few as one per nematode in the case of *Xenorhabdus* reassociations (Stilwell et al., 2018). Moreover, a highly species-specific reassociation occurs in *Xenorhabdus*-*Steinernema* complexes, and in *Xenorhabdus nematophila*, this is attributed to the NilC protein (Cowles and Goodrich-Blair, 2004). By retaining the second larval stage cuticle, J3s complete their transformation to IJ3s that then emigrate the cadaver in search of new insect prey. Notably, all seven macrocyclic antimicrobial non-ribosomal peptides (NRPs) with known toxicities—chaiyaphumins, photoditritide, szentiamide, xenobactin, phototemtide, xenoamicin, and PAX lipopeptides—were lowly toxic to mammalian cells—the lowest half-maximal inhibitory concentration (IC_50_) was 52 μM (Gualtieri et al., 2009; Ohlendorf et al., 2011; Grundmann et al., 2013, 2014; Zhou et al., 2013; Zhao et al., 2019, 2020). The bacteria possibly evolved to synthesize these compounds to inhibit diverse soil microorganisms while remaining lowly toxic to animal nematode hosts (Racine and Gualtieri, 2019). Biotechnologically, their low toxicity, natural derivatization, and macrocyclic structure (Dathe et al., 2004; Rodríguez et al., 2021) make them suitable for antibiotic drug development.

## *Xenorhabdus*/*Photorhabdus* molecules in drug development

Many *Xenorhabdus/Photorhabdus* molecules have the potential to be developed into approved drugs (Challinor and Bode, 2015; Dreyer et al., 2018; Racine and Gualtieri, 2019; Booysen and Dicks, 2020). For example, *Photorhabdus luminescens* biosynthesized 3,5-dihydroxy-4-isopropylstilbene (Hu et al., 1997)—this is the active pharmaceutical ingredient in the drugs benvitimod and tapinarof (Zhang et al., 2022), which are approved for market in China and the USA, respectively, for the treatment of psoriasis and topical dermatitis (Lebwohl et al., 2021). NOS-502, an antibiotic lead compound currently in pre-clinical development, is a synthetic derivative of the odilorhabdins (Figure 1). These are cationic antimicrobial NRPs biosynthesized by *X. nematophila* that inhibit protein synthesis *via* unique sites on the 30S ribosome (Pantel et al., 2018). NOS-502 not only had a good *in vivo* safety profile but also inhibited beta-lactam resistant strains of both *Escherichia coli* and *Klebsiella pneumoniae* at minimum inhibitory concentrations (MICs) of 1.85 and 0.93 μM, respectively (Zhao M. et al., 2018). Another lead compound in pre-clinical development is darobactin A which is produced by *Photorhabdus khanii* (Lewis, 2020). It too was lowly toxic in murine models and inhibited beta-lactam resistant strains of both *E. coli* and *K. pneumoniae* at an MIC of 2.1 μM (Imai et al., 2019).

The development of novel antimalarial drugs is of current global health importance due to increasing resistance to artemisinin-based therapies in malaria-endemic regions such as East Africa (Asua et al., 2021), because of mutations in the *Plasmodium falciparum K13* gene (Amaratunga et al., 2019). Two potential antimalarial lead compounds for pre-clinical development are the NRPs chaiyaphumin A from *Xenorhabdus* sp. PB61.4 (Grundmann et al., 2014) and rhabdopeptide/xenortide-like peptide (RXP) 6 from *Xenorhabdus innex*i (Zhao L. et al., 2018). This is because RXP 6 and chaiyaphumin A were inhibitory to *P. falciparum* at IC_50_ of 0.091 and 0.61 μM, respectively. Moreover, they had respective selectivity indexes of 63 and 151. Biochemically, the bioactivity of chaiyaphumin A was affected by the fatty acid acylated to its *N* terminal as the natural swapping of phenylacetic acid for n-butyrate created a derivative with an IC_50_ of 15.4 μM and selectivity index of 10. Thus, a probable route for creating chaiyaphumin derivatives with enhanced pharmacological properties is by swapping the C_starter_ domain of its non-ribosomal peptide synthetase (NRPS) *via* NRPS re-engineering (Beck et al., 2020).

Although antibody–drug conjugates are promising anticancer therapies, their intrinsic high cost of development makes the price of approved drugs—such as enfortumab vedotin for the treatment of urothelial carcinoma—currently cost-ineffective (Wu et al., 2022). A possible solution is replacing the antibody component with a modified *Photorhabdus* Tc toxin, to translocate—within a cocoon-like structure (Roderer et al., 2019)—and deliver cytotoxic compounds into targeted cancer cells (Nǵanǵa et al., 2019). However, the concept that the *Photorhabdus* TcA subunit can selectively bind to a cancer cell needs to be first proven.

## Conclusion

Twenty-seven *Xenorhabdus*, twenty-one *Photorhabdus* species, and four *Serratia* strains were identified as isolated from EPNs. Sixty-three novel species of *Xenorhabdus* could be discovered from corresponding characterized but under-investigated steinernematids. Due to the low phylogenetic diversity in the genus, the use of pangenome analyses for species delineation in *Photorhabdus* is recommended when enough genomes per species are available. The lifecycle of the nematode-bacterium complex is marked by the secretion of diverse bioactive bacterial molecules in the presence of juvenile nematodes, necessitating high selectivity. Thus, many of these molecules are applicable in biotechnology and a few are currently in drug development pipelines. This highlights the practical importance of discovering more nematophilic bacteria: They are a source of novel therapeutics.

## Author contributions

RA did the research, wrote the manuscript, and approved the submitted version.
